# Supplementary material for: The mitochondrial inner membrane protein LETM1 modulates cristae organization through its LETM domain
Source: Commun Biol. 2020 Mar 5;3:99. doi: 10.1038/s42003-020-0832-5 (PMC7058069; doi:10.1038/s42003-020-0832-5)
Supplement: Supplementary file 1 — Supplementary information [file 42003_2020_832_MOESM1_ESM.pdf]

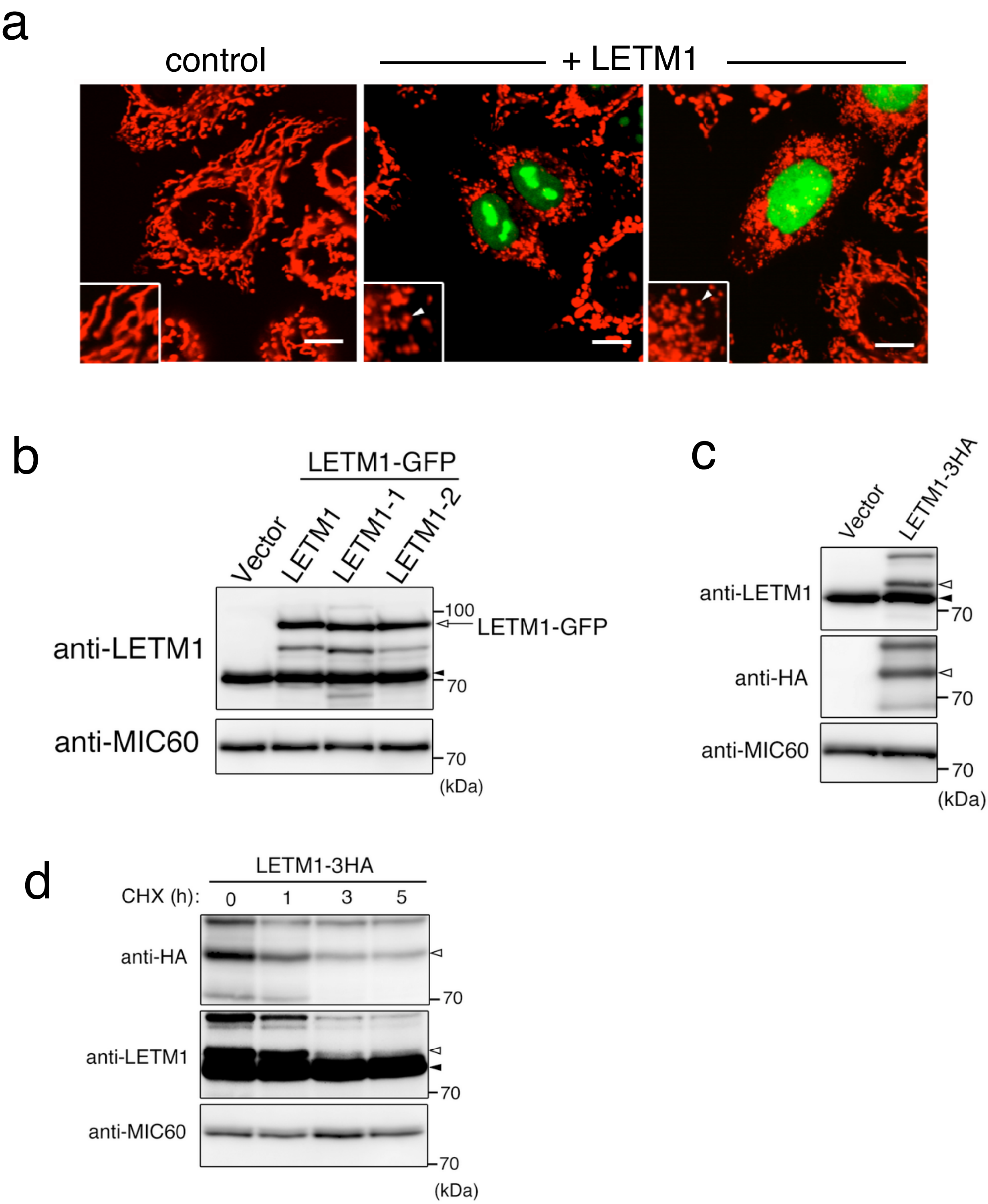

Supplementary Figure 1 Elevated expression of LETM1 induces mitochondrial fragmentation. **a** HeLa cells stably expressing a mitochondria-targeted red fluorescent protein were transfected with plasmid coexpressing LETM1 and nuclear-targeted GFP. Live images of untransfected (control) and transfected cells (+ LETM1) were obtained by confocal microscopy. High-magnification images are superimposed. Arrowheads indicate fragmented mitochondria. Scale bar, 5 μm. **b** Cell lysates were prepared from cells transfected with vector and expression plasmids carrying wild-type or mutant LETM1-GFP and analyzed by immunoblotting using antibody to either LETM1, or MIC60 as a loading control. Arrowhead and arrow indicate LETM1-GFP and endogenous LETM1 protein, respectively. **c** Cell lysates were prepared from cells transfected with vector and expression plasmids carrying LETM1-3HA and subjected to SDS-PAGE and immunoblotting using antibody to either HA tag, LETM1, or MIC60 as a loading control. Solid and open arrowheads represent endogenous LETM1 and LETM1-3HA proteins, respectively. **d** LETM1-3HA expressing cells were cultured for the indicated times after adding 0.1 mg/ml cycloheximide (CHX). Cell lysates were prepared and subjected to SDS-PAGE and immunoblotting using antibody to either HA tag, LETM1, or MIC60 as a loading control.

e

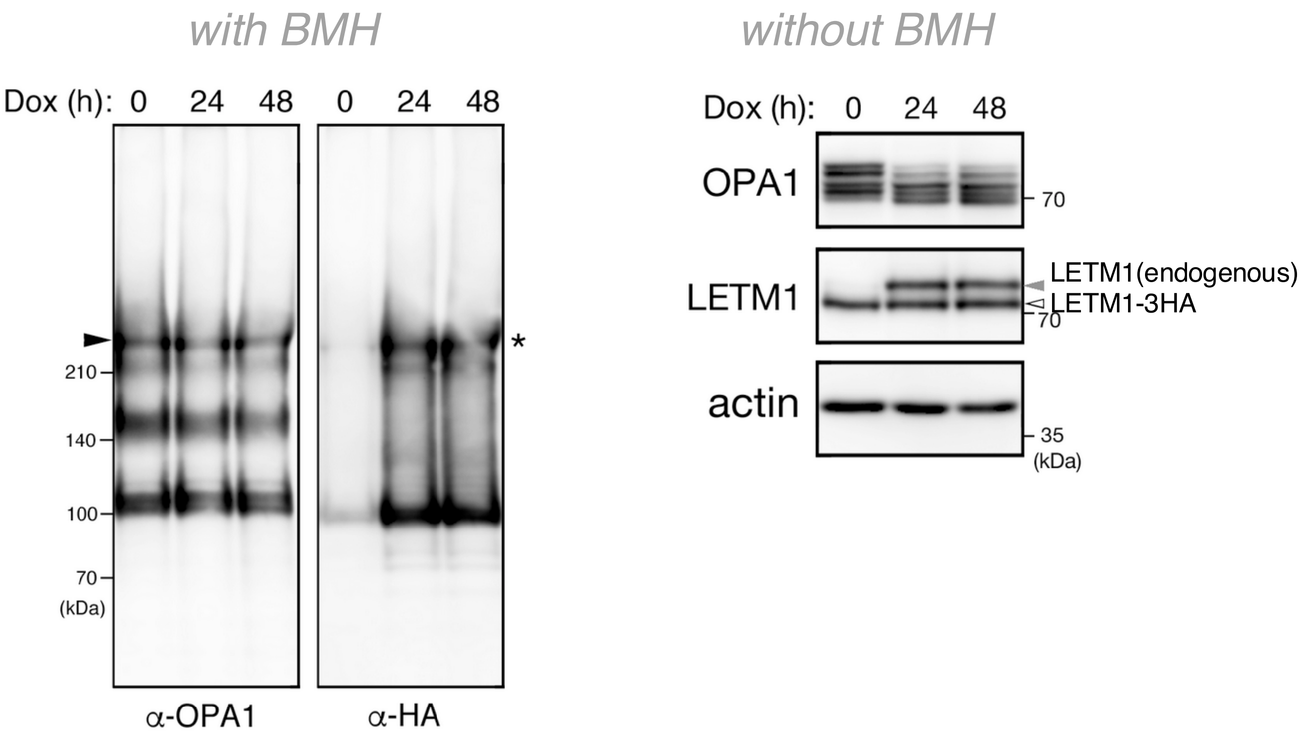

f

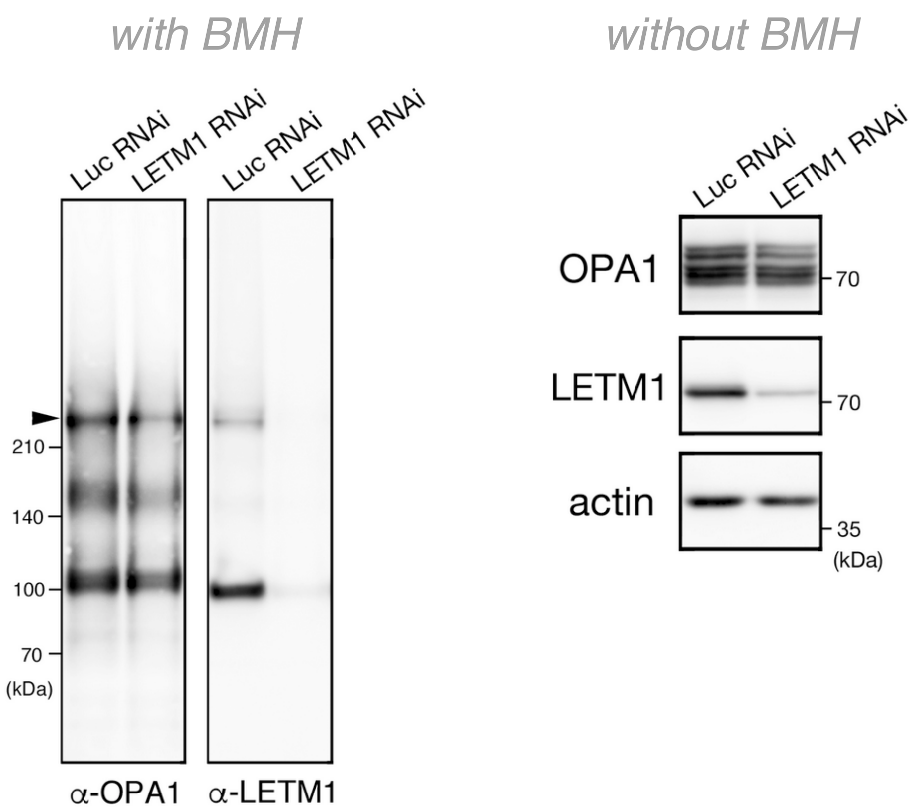

Supplementary Figure 1. **e** HEK cell lines stably expressing LETM1-3HA were cultured in medium containing 1 μg/ml doxycycline (Dox) for the indicated times. Cells were harvested and divided into two populations: *With BMH;* cells were subjected to treatment with a membrane-permeable cross linker, BMH and subsequently analyzed by NuPAGE and immunoblot using antibodies to either OPA1, or HA tag. Black arrowhead and asterisk indicate cross-linked oligomers of OPA1 and LETM1-3HA, respectively. *Without BMH*; another population was subjected to analysis by SDS-PAGE and immunoblot without treatment with BMH. Gray and open arrowheads indicate LETM1-3HA proteins and endogenous LETM1, respectively. **f** Cells transfected with either control siRNA (Luc) or siRNA for LETM1 were harvested and divided into two populations. *With BMH*; cells were treated with BMH and the cell lysates were analyzed by NuPAGE and immunoblot using antibodies to either OPA1, or LETM1. Arrowhead indicates cross-linked oligomers of OPA1. *Without BMH*; another population was directly subjected to SDS-PAGE and immunoblot using antibodies to OPA1, LETM1, or actin as a loading control.

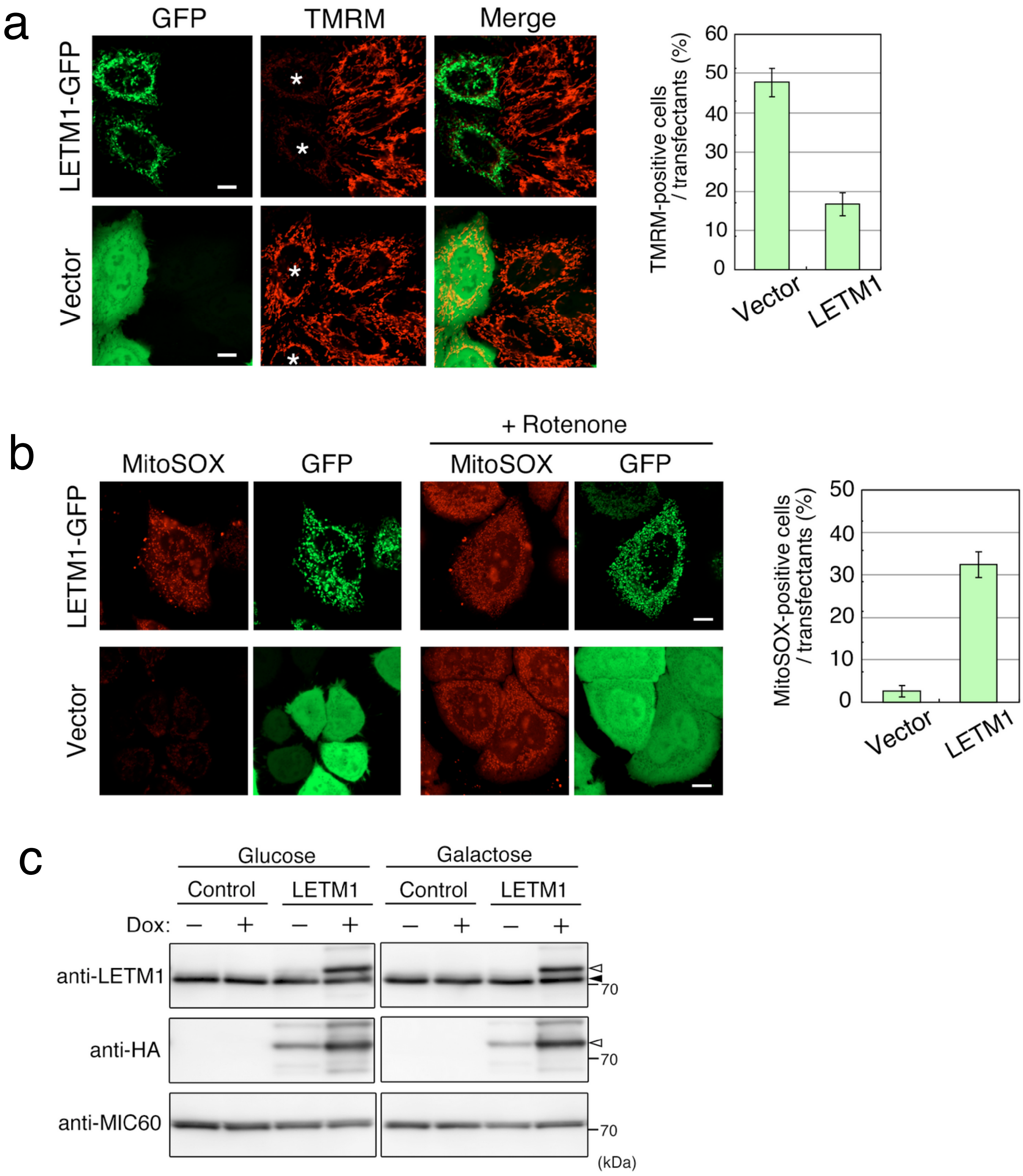

Supplementary Figure 2. Effects of increased LETM1 expression on mitochondrial membrane potential and cell growth. **a** HeLa cells were transfected with a plasmid carrying GFP (Vector) or LETM1-GFP and stained with a membrane potential-dependent fluorescent dye, TMRM. Live images were obtained by confocal microscopy. Asterisks indicate LETM1-GFP-expressing cells. Scale bar, 10 μm. Data represent the mean ± SEM of three independent experiments; >100 individual transfected cells were counted. **b** HeLa cells were transfected with a plasmid carrying GFP (Vector) or LETM1-GFP and stained with a fluorescent superoxide indicator, MitoSOX Red, to monitor mitochondrial reactive oxygen species (ROS). Live images were obtained by confocal microscopy. Rotenone was used for positive control experiments. Scale bar, 10 μm. Data represent the mean ± SEM of three independent experiments; >100 individual transfected cells were counted. **c** HEK cell lines stably expressing either chloramphenicol acetyltransferase (control) or LETM1-3HA were cultured in medium containing the indicated sugars in the presence or absence of 1 μg/ml doxycycline (Dox). Cell lysates were prepared and subjected to SDS-PAGE and immunoblotting using antibodies to either HA tag, LETM1, or MIC60 as a loading control. Solid and open arrowheads indicate endogenous LETM1 and LETM1-3HA proteins, respectively.

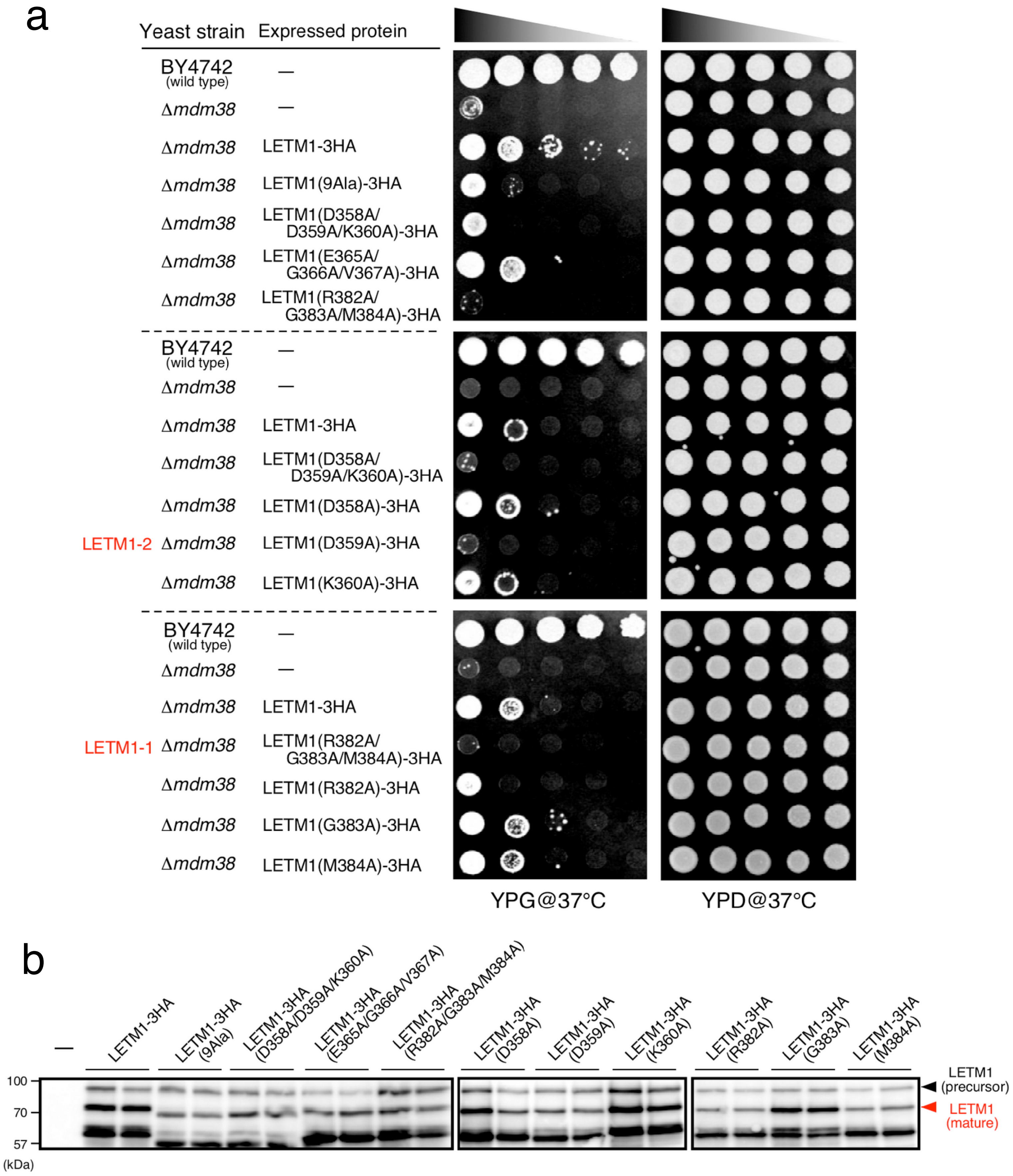

Supplementary Figure 3. A screen for the amino acid residues in the LETM domain required for growth complementation in yeast *mdm38* mutants. **a** Growth phenotypes of yeast *mdm38* mutant cells expressing LETM1 mutants. Serial dilutions of wild-type (BY4742) and Δ*mdm38* mutant cells expressing the indicated proteins were plated at 37°C onto rich medium containing either glycerol (YPG) or dextrose (YPD).
b Cell lysates were prepared from yeast cells expressing the indicated proteins and subjected to SDS-PAGE and immunoblotting using antibody to HA tag. Black and red arrowheads indicate the precursor and mature forms of LETM1-3HA, respectively.

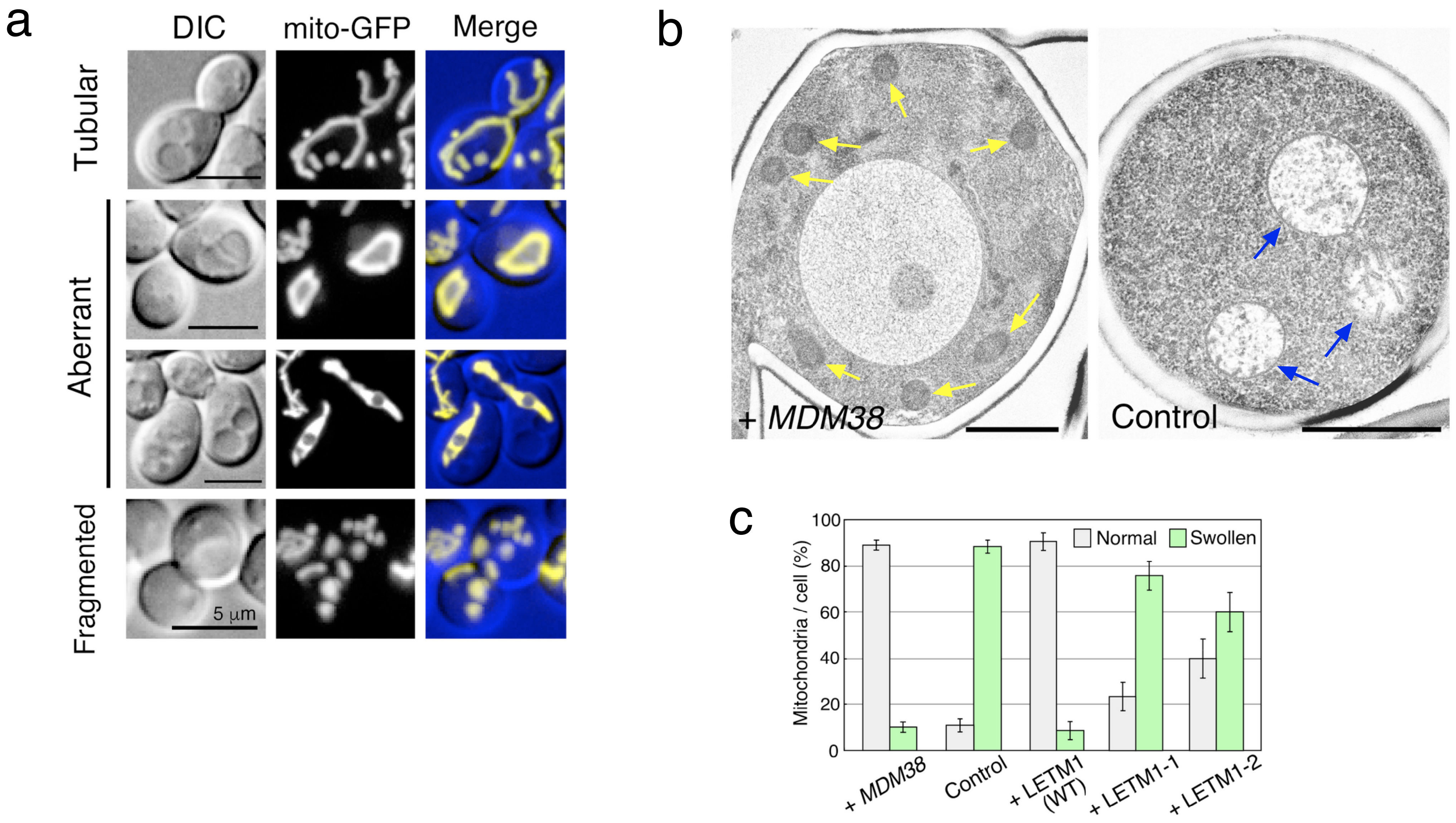

Supplementary Figure 4. Analysis of mitochondrial morphology of yeast *mdm38* mutant cells. **a** Live fluorescent images of Δ*mdm38* cells carrying mitochondria-targeted GFP were obtained by confocal microscopy and classified into three categories (tubular, aberrant, and fragmented). Scale bar, 5 μm. **b** Δ*mdm38* mutant cells carrying a plasmid containing a yeast *MDM38* gene or control plasmid were fixed, and thin sections were visualized by electron microscopy. Yellow and blue arrowsindicate normal and swollen mitochondria, respectively. Scale bar, 1 μm. **c** Mitochondrial morphology of 25 individual cells in the thin sections was scored. Data represent the mean ± SEM.

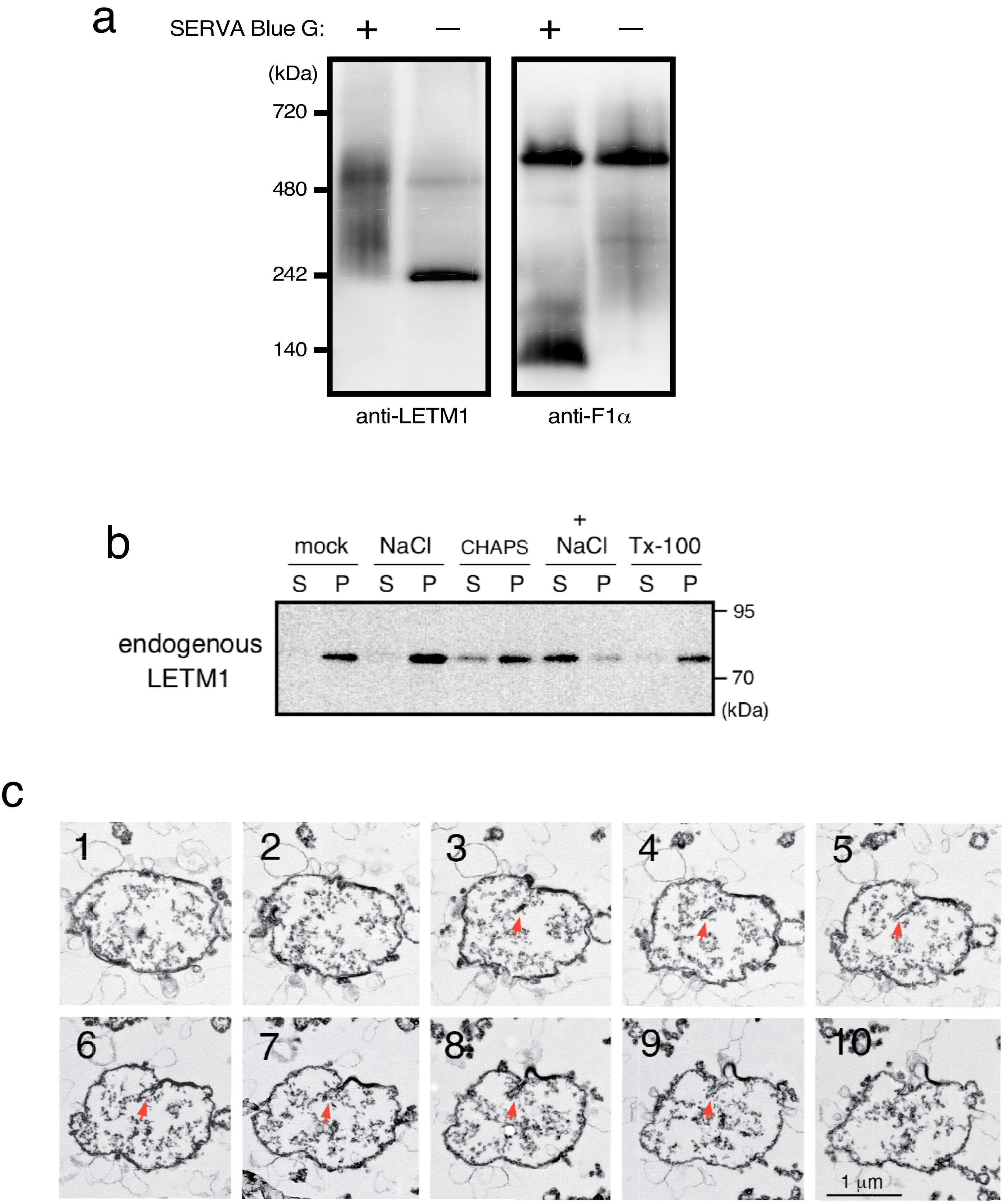

Supplementary Figure 5. LETM1 facilitates *in vitro* membrane invagination. **a** Extraction of endogenous LETM1 protein with detergents. Mitochondria-enriched fractions prepared from HeLa cells were treated with the indicated detergents in the absence or presence of 100 mM NaCl and subjected to immunoblotting using anti-LETM1 antibody. **b** Digitonin-solubilized mitochondrial fractions were analyzed by clear-native PAGE in the absence or presence of dye (SERVA Blue G) and subjected to immunoblotting using anti-LETM1 antibody. **c** Electron microscopic images of serial sections of a proteoliposome containing wild-type His-LETM1. Arrows indicate membrane invagination. These images were used for the three-dimensional reconstitution shown in Fig. 4d and Supplementary Movie 1.

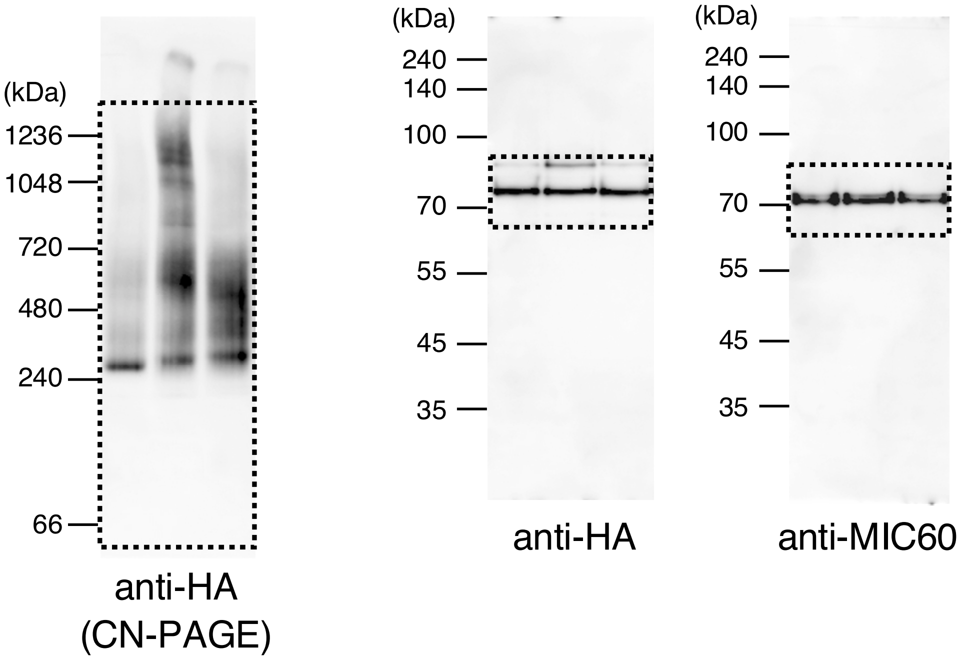

Supplementary Figure 6. Images of uncropped immunoblots for Fig. 3c.
