# Supplementary material for: The mitochondrial inner membrane protein LETM1 modulates cristae organization through its LETM domain
Source: Commun Biol. 2020 Mar 5;3:99. doi: 10.1038/s42003-020-0832-5 (PMC7058069; doi:10.1038/s42003-020-0832-5)
Supplement: Supplementary file 2 — Description of Additional Supplementary Files [file 42003_2020_832_MOESM2_ESM.pdf]

Description of additional supplementary items

Supplementary Data1 Source data of this study.

The source data underlying plots shown in this paper was provided.

Supplementary Movie 1. A movie of three-dimensional images reconstituted from serial sections of the proteoliposome containing wild-type His-LETM1 shown in Supplementary figure 5c.
