# Supplementary material for: The mitochondrial inner membrane protein LETM1 modulates cristae organization through its LETM domain
Source: Commun Biol. 2020 Mar 5;3:99. doi: 10.1038/s42003-020-0832-5 (PMC7058069; doi:10.1038/s42003-020-0832-5)
Supplement: Supplementary file 5 — Reporting Summary [file 42003_2020_832_MOESM5_ESM.pdf]

nature research

Corresponding author(s): Toshihiko Oka

Last updated by author(s): 1/27/2020

Reporting Summary

Nature Research wishes to improve the reproducibility of the work that we publish. This form provides structure for consistency and transparency in reporting. For further information on Nature Research policies, see Authors & Referees and the Editorial Policy Checklist.

Statistics

For all statistical analyses, confirm that the following items are present in the figure legend, table legend, main text, or Methods section.

n/a	Confirmed	
☐	☒	The exact sample size (*n*) for each experimental group/condition, given as a discrete number and unit of measurement
☐	☒	A statement on whether measurements were taken from distinct samples or whether the same sample was measured repeatedly
☐	☒	The statistical test(s) used AND whether they are one- or two-sided<br>*Only common tests should be described solely by name; describe more complex techniques in the Methods section.*
☒	☐	A description of all covariates tested
☒	☐	A description of any assumptions or corrections, such as tests of normality and adjustment for multiple comparisons
☐	☒	A full description of the statistical parameters including central tendency (e.g. means) or other basic estimates (e.g. regression coefficient) AND variation (e.g. standard deviation) or associated estimates of uncertainty (e.g. confidence intervals)
☐	☒	For null hypothesis testing, the test statistic (e.g. F, t, r) with confidence intervals, effect sizes, degrees of freedom and P value noted<br>*Give P values as exact values whenever suitable.*
☒	☐	For Bayesian analysis, information on the choice of priors and Markov chain Monte Carlo settings
☒	☐	For hierarchical and complex designs, identification of the appropriate level for tests and full reporting of outcomes
☒	☐	Estimates of effect sizes (e.g. Cohen's *d*, Pearson's *r*), indicating how they were calculated

Our web collection on statistics for biologists contains articles on many of the points above.

Software and code

Policy information about availability of computer code

Data collection	*Provide a description of all commercial, open source and custom code used to collect the data in this study, specifying the version used OR state that no software was used.*
Data analysis	*Provide a description of all commercial, open source and custom code used to analyse the data in this study, specifying the version used OR state that no software was used.*

For manuscripts utilizing custom algorithms or software that are central to the research but not yet described in published literature, software must be made available to editors/reviewers. We strongly encourage code deposition in a community repository (e.g. GitHub). See the Nature Research guidelines for submitting code & software for further information.

Data

Policy information about availability of data

All manuscripts must include a data availability statement. This statement should provide the following information, where applicable:

- Accession codes, unique identifiers, or web links for publicly available datasets
- A list of figures that have associated raw data
- A description of any restrictions on data availability

All the data used in this study are available within the manuscript and its Supplementary Information files or from the corresponding author upon reasonable request.

Field-specific reporting

Please select the one below that is the best fit for your research. If you are not sure, read the appropriate sections before making your selection.

☒ Life sciences ☐ Behavioural & social sciences ☐ Ecological, evolutionary & environmental sciences

For a reference copy of the document with all sections, see nature.com/documents/nr-reporting-summary-flat.pdf

Life sciences study design

All studies must disclose on these points even when the disclosure is negative.

Sample size	We used either more than 100 mammalian cells, more than 50 yeast cells, or more than 20 mitochondrial sections per experiment for quantitative analyses, and more than 50 liposemes per experiment were applied to analyze in vitro membrane invagination. All data were obtained from three independent experiments to correct for the variations.
Data exclusions	No data were excluded from the analyses.
Replication	The reproducibility of all experimental findings in this study was confirmed.
Randomization	The samples were randomly selected.
Blinding	The study did not contain Human research and the samples were randomly selected. Therefore, blinding was not relevant.

Reporting for specific materials, systems and methods

We require information from authors about some types of materials, experimental systems and methods used in many studies. Here, indicate whether each material, system or method listed is relevant to your study. If you are not sure if a list item applies to your research, read the appropriate section before selecting a response.

Materials & experimental systems

n/a	Involved in the study	
☐	☒	Antibodies
☐	☒	Eukaryotic cell lines
☒	☐	Palaeontology
☒	☐	Animals and other organisms
☒	☐	Human research participants
☒	☐	Clinical data

Methods

n/a	Involved in the study	
☒	☐	ChIP-seq
☒	☐	Flow cytometry
☒	☐	MRI-based neuroimaging

Antibodies

Antibodies used	The following primary antibodies were used: LETM1 (Abnova, clone 6F7), Tom20 (Santa Cruz Biotechnology, clone F-10), mtHSP70 (Stressgen, clone BB70), cytochrome c (BD Pharmingen, clone 6H2.B4), MIC60 (Invitrogen, clone 2E4AD5), F1a (Invitrogen, clone 15H4C4), OPA1 (BD Biosciences, clone 18), actin (Wako Chemicals, Cat#013-24553), HA tag (Roche, clone 3F10), and HA tag (Wako Chemicals, clone 4B2).
Validation	LETM1 (Abnova, clone 6F7): http://www.abnova.com/products/products_detail.asp?catalog_id=H00003954-M03<br>Tom20 (Santa Cruz Biotechnology, clone F-10): https://www.scbt.com/scbt/product/tom20-antibody-f-10?productCanUrl=tom20-antibody-f-10&_requestid=300953<br>mtHSP70 (Stressgen, clone BB70): http://www.merckmillipore.com/JP/en/product/Anti-mtHsp70-Antibody-clone-JG1,MM_NF-MABS1955-100UL?ReferrerURL=https%3A%2F%2Fwww.google.com%2F&bd=1<br>cytochrome c (BD Pharmingen, clone 6H2.B4): http://www.bdbiosciences.com/eu/applications/research/apoptosis/purified-antibodies/purified-mouse-anti-cytochrome-c-6h2b4/p/556432<br>MIC60 (Invitrogen, clone 2E4AD5): https://www.thermofisher.com/antibody/product/Mitofilin-Antibody-clone-2E4AD5-Monoclonal/45-6400<br>F1a (Invitrogen, clone 15H4C4): https://www.thermofisher.com/antibody/product/ATP5A1-Antibody-clone-15H4C4-Monoclonal/43-9800<br>OPA1 (BD Biosciences, clone 18): https://www.bdbiosciences.com/eu/reagents/research/antibodies-buffers/cell-biology-reagents/cell-biology-antibodies/purified-mouse-anti-opa1-18opa1/p/612606<br>actin (Wako Chemicals, Cat#013-24553): https://labchem-wako.fujifilm.com/jp/product/detail/W01W0101-2784.html<br>HA tag (Roche, clone 3F10): https://www.sigmaaldrich.com/catalog/product/roche/roahaha?lang=en&region=US<br>HA tag (Wako Chemicals, clone 4B2): https://labchem-wako.fujifilm.com/us/product/detail/W01W0101-2188.html

Eukaryotic cell lines

Policy information about cell lines

Cell line source(s)	HeLa cells, HeLa cells expressing stably mitochondria-targeted RFP, and HEK cell line expressing stably LETM1-3HA
Authentication	HeLa and HEK cells were purchased from ATCC and Clontech, respectively. The stable cell lines used in the study were derived from the corresponding parental cells.
Mycoplasma contamination	All cell lines tested were negative for mycoplasma comtamination.
Commonly misidentified lines (See ICLAC register)	No commonly misidentified lines were used.
